# Heart rate variability in bipolar disorder and borderline personality disorder: a clinical review

**DOI:** 10.1136/eb-2017-102760

**Published:** 2018-01-23

**Authors:** Oliver Carr, Maarten de Vos, Kate E A Saunders

**Affiliations:** 1 Institute of Biomedical Engineering, University of Oxford, Oxford, UK; 2 University of Oxford Department of Psychiatry, Warneford Hospital, Oxford, UK; 3 Oxford Health NHS Foundation Trust, Warneford Hospital, Oxford, UK

**Keywords:** cardiology, pacing & electrophysiology, psychiatry, depression & mood disorders, personality disorders

## Abstract

Heart rate variability (HRV) in psychiatric disorders has become an increasing area of interest in recent years following technological advances that enable non-invasive monitoring of autonomic nervous system regulation. However, the clinical interpretation of HRV features remain widely debated or unknown. Standardisation within studies of HRV in psychiatric disorders is poor, making it difficult to reproduce or build on previous work. Recently, a Guidelines for Reporting Articles on Psychiatry and Heart rate variability checklist has been proposed to address this issue. Here we assess studies of HRV in bipolar disorder and borderline personality disorder against this checklist and discuss the implication for ongoing research in this area.

## Introduction

**Figure 1:**
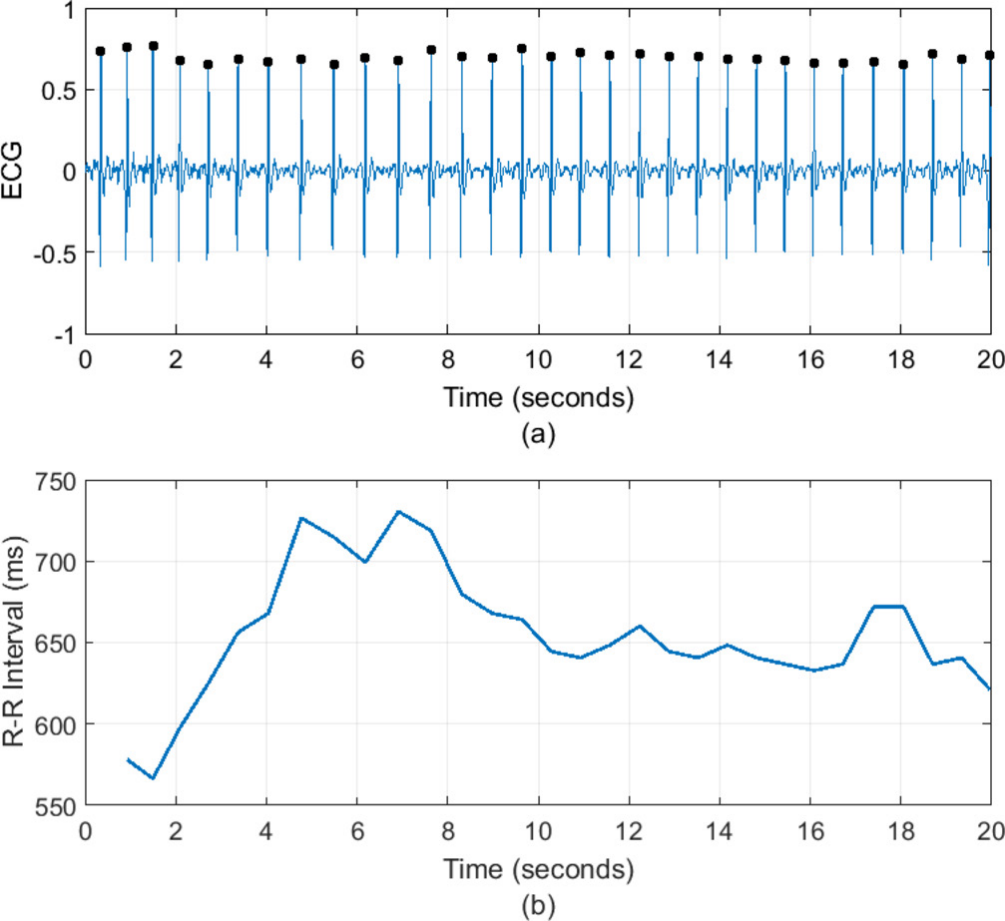
(A) An ECG signal with R peaks denoted by black dots. (B) The corresponding tachogram or R–R interval series.

Heart rate variability (HRV) is the variation in time interval between each heart beat, recorded as the R–R interval of an ECG signal. It is a complex physiological phenomenon that results from the modification of the heart rate (HR) by respiratory, circulatory, autonomic, endocrine and mechanical factors. Reductions in HRV are associated with a range of conditions such as diabetic neuropathy, sepsis and following myocardial infarct but have become of increasing interest in psychiatry because of the link between autonomic dysfunction and psychiatric illness. Changes in HRV have been reported in a range of mental disorders^1^ as well as correlating with psychological dimensions such as social cognition,^2^ executive function^3^ and emotional regulation.^4^ The higher incidence of cardiovascular disease associated with some psychiatric disorders has also led to greater focus on autonomic system function. Both bipolar disorder (BD) and borderline personality disorder (BPD) have higher rates of cardiovascular mortality. Despite their contrasting aetiologies and treatments, they are phenotypically similar, both being characterised by mood instability. This makes them ideal groups in which to compare HRV (figure 1).

Variations in HR occur due to the constant need of the heart to adapt to changing circumstances, and it is thought that loss of balance of the sympathetic and parasympathetic nervous systems causes an alteration to the structure of HRV.^5 6^ HRV, as a measure of nervous system balance, can therefore provide a quantification of physiological changes associated with mental health disorders, with many studies investigating these associations through a number of time domain, frequency domain and non-linear methods of quantifying HRV.^7–9^

Initial studies of HRV used relatively simple linear algorithms to quantify variability and autonomic function, either in the time domain or the frequency domain.^7^ Later studies suggested that interactions between the autonomic nervous system and the regulation of the cardiovascular system may be non-linear, with more complex non-linear algorithms potentially providing better metrics to quantify these interactions. In addition, non-linear measures are often less affected by non-stationarity of signals, whereas linear methods require stationary signals.^10^

The linear time domain features are the most straightforward metrics of HRV to calculate. These measures are statistical calculations of the intervals between successive normal complexes (see table 1). Frequency domain features are derived using spectral analysis. This approach provides information about how the variance (or power) is distributed as a function of frequency. These tend to be applied either to short stationary recordings (5 min) or over 24-hour periods, with frequency metrics being linked to levels of sympathetic and primarily parasympathetic activity in the autonomic nervous system (ANS).^11^

**Table 1:** The most widely used HRV measures used in the literature with short interpretation of their meanings

HRV measure	Units	Domain	Description
Mean of R–R intervals (mRR)	ms	Time domain	A measure of average R–R interval (60/heart rate).
Standard deviation (SD) of R–R intervals (SDNN)	ms	Time domain	A measure of variability of R–R intervals across the whole signal.
Root mean square of successive differences	ms	Time domain	A measure of shorter term variation through differences between adjacent R–R intervals.
SD of successive differences	ms	Time domain	A longer term measure of variability through SD of differences between adjacent R–R intervals.
SD of average R–R intervals	ms	Time domain	For longer signals, mRR is calculated on segments, often 5 min, and SD of these values are calculated.
Number of R–R intervals over x ms	–	Time domain	Often R–R intervals over 50 ms, count of longer intervals to determine variability.
Percentage of R–R intervals over x ms	%	Time domain	As above, normalised to the total number of intervals. Can be used when signal lengths vary.
Total power (TP)	ms^2^	Frequency domain	Total power in the frequency spectra up to 0.4 Hz. Can be measured from zero, from the start of the VLF band (0.003 Hz) or from the LF band (0.04 Hz).
Very low frequency power (VLF)	ms^2^	Frequency domain	Power in the 0.003–0.04 Hz frequency band.
Low frequency power (LF)	ms^2^	Frequency domain	Power in the 0.04–0.15 Hz frequency band. Often linked to combined levels of sympathetic and parasympathetic activity; however, this interpretation is widely debated.
High frequency power (HF)	ms^2^	Frequency domain	Power in the 0.15–0.4 Hz frequency band. Linked to levels of parasympathetic activity and frequencies of respiration.
Normalised low frequency power (nLF) and normalised HF power (nHF)	–	Frequency domain	Power in each frequency band normalised to the total power.
Low frequency to high frequency (LF/HF) ratio	–	Frequency domain	Ratio between LF and HF power bands. Often associated with sympathovagal balance in the literature; however, this interpretation is also debated.
Sample entropy	–	Non-linear	Entropy measures periodic variations in the R–R interval signals not detectable using means and SD.
Detrended fluctuation analysis exponent (α)	–	Non-linear	Finds long-term correlations in the signal, with the exponent giving a value of self-correlation of the signal.
Poincaré standard deviations (SD1, SD2)	–	Non-linear	Poincaré plots plot R–R intervals against the succeeding R–R intervals. With the SD in y=x representing longer term variation and in the perpendicular direction, short-term variation.
pNN50	-	Time domain	Proportion of consecutive R-R intervals that differ by more than 50ms. Measure of parasympathetic activity.

HRV, heart rate variability.

Non-linear features that have become more widely used include: measures of *entropy* (sample entropy and approximate entropy), which measure irregularity and randomness of signals; *detrended fluctuation analysis*, to distinguish between short-term internal variations and longer term variations; *power law exponent*, which determines the fractal nature of the interbeat interval signal; and *recurrence quantification analysis*, a method to quantify repeating instances of signals, as well as algorithms such as *Teager-Kaiser energy* and *Lempel-Ziv complexity*. Although these non-linear methods of quantifying HRV may give more insight into the complex interactions linked to the cardiovascular system, it is much more difficult to give clinical interpretation to their metrics.^8 12 13^

Studies involving HRV in psychiatry illness have minimal standardisation, especially in the timescales and methods of recording ECG data. The majority of studies obtain ECG recordings from a 5 min period and calculate HRV measures on the 5 min R–R interval signal. However, as much longer recordings are becoming easier to obtain, HRV measures can be calculated over much longer periods, making comparison between HRV measures on different timescales difficult. In addition to the varying timescales of recordings, the activity levels of the participants and method of data collection may also differ. For example, data are often collected when the participant is ‘at rest’; however, the posture of the participant, time of day, recently eating or drinking and many other factors may all contribute to variations in HRV. Stimuli, in the form of images or exercises, may also be given during the recordings, again making comparison of HRV measures between studies difficult. Development of devices allowing ambulatory monitoring of ECG over periods of hours or even days enable participants to continue with their regular activity and behavioural patterns while being recorded. While these data can be recorded in a less clinical and more natural manner, it becomes increasingly difficult to monitor all external factors that considerably alter the behaviour of the cardiovascular system. For studies involving mental health or investigating difference in HRV between groups, it is often not possible to determine whether differences are due to the different diagnoses or due to the external influences.

There are no widely agreed standard measures for HRV quantification and the clinical interpretation of many HRV features remain widely debated or unknown. Quintana *et al*^14^ provided recommendations to improve HRV research in psychiatry and introduced a Guidelines for Reporting Articles on Psychiatry and Heart rate variability (GRAPH) checklist for good practice to make an attempt to standardise reporting in these studies. The guidelines consist of four main areas: selection of participants, interbeat interval collection, data analysis and cleaning and HRV calculation. Within each topic area a list of checklist items is defined, as shown in table 2.

**Table 2:** Guidelines for Reporting Articles on Psychiatry and Heart rate variability^14^

Topic	Number	Checklist Item
Participant selection	1	
Psychiatric group selection	1a	Recruitment details and assessment methods
Control group selection	1b	Recruitment details and methods to rule out psychiatric illness
Inclusion criteria	1c	Description of criteria (eg, absence of physical health problems)
Disease characteristics	1d	Description of disease duration, severity, comorbidities and medication
Demographics	1e	Details on age, gender, physical activity levels, smoking and so on
R–R interval collection	2	
Hardware/software details	2a	Brand and electrode configuration
R–R interval collection details	2b	Sampling rate, length of data, time of day, filtering, posture and participant instructions
Data analysis and cleaning	3	
R–R interval calculation	3a	R–R interval calculation and resampling methods
R–R interval artefact identification	3b	R–R interval artefact identification method (eg, algorithm and manual inspection)
R–R interval data loss	3c	Reasons for loss (eg, ectopy and equipment failure)
R–R interval cleaning	3d	Artefact cleaning methods and percentage of corrected beats
HRV calculation	4	
Method of analysis used	4a	Metrics used and software for calculation, log transform
Frequency bands used	4b	Specification of frequency bands and their interpretation

HRV, heart rate variability.

Here we review HRV in BD and BPD making specific reference to the good practice checklist. BPD and BD are two psychiatric diagnoses that despite differing aetiologies have a shared phenotype; both disorders are associated with mood instability, impulsivity, suicidal behaviour and low mood. Comparing HRV in the two disorders allows us to explore whether the shared phenotype is associated with similar or differing underlying physiology.

## Methods

To identify relevant evidence of HRV in mood disorders, we searched PubMed, PsycINFO, Google Scholar and the Cochrane Library for papers published between 1980 and May 2017. No language constraints were applied, and the following key words were used: depression, bipolar disorder, borderline personality disorder and heart rate variability. This search was supplemented by hand search of references from articles in the initial search. Articles were included in which ECG recordings were analysed from at least one participant diagnosed with either BD or BPD.

The methods, results and interpretation of each article were summarised, and their adherence to the GRAPH checklist was examined.

## Presentation

### HRV in BD

A range of studies of HRV in patients suffering from BD have been conducted but are often difficult to compare due to the BD participants being in varying mood states across the studies. Statistical comparisons are often made between the psychiatric disorder groups and a control group and occasionally between participants in different mood states. The earliest study was carried out by Cohen *et al.*^7^ ECG from 39 euthymic bipolar patients and 39 healthy controls were recorded in a controlled environment. Time domain and spectral analysis were performed on the R–R intervals, with bipolar patients having lower root mean square of successive differences (RMSSD), lower total power, higher nHF and an increased low frequency to high frequency (LF/HF) ratio, independent of any medication taken. The features calculated were defined slightly differently to the standard methods and with no information given about the length of time of ECG recording thus making comparisons with this study difficult. Further work was carried out on the same participants in 2005 by Todder *et al* using non-linear analysis; however, they found no significant differences between the euthymic BD and healthy groups using the non-linear features that include: Lyapunov exponent, Shannon entropy and Poincaré plots.^15^

Other studies carried out on euthymic bipolar patients compared with healthy controls include the study by Gruber *et al* in 2011. Here, the emotional responses of 23 BD participants were compared with that of 24 healthy controls after various stimuli; the study found greater HRV in the BD group after the stimuli through an increase in measures related to parasympathetic activity.^16^ More recently, in 2015, Voggt *et al* investigated HRV features in 90 euthymic bipolar patients compared with 62 healthy controls. Parameters were calculated from a 30 min ECG recording, with SD of R–R interval (SDNN), low frequency (LF) power and high frequency (HF) power found to be lower in the BD group.^17^ In 2012, Levy used several physiological measures of autonomic nervous system function to determine differences between patients with BD and healthy controls, without using the traditional HRV features. Thirty-three patients with BD and 22 healthy controls had 5 min of ECG recorded at rest, with significant differences found in physiological HR features between patients with BD and healthy controls.^18^

Lee *et al*^19^ investigated the differences in HRV between 33 BD patients with subsyndromal depressive symptoms and 59 healthy controls. A 5 min section of ECG was recorded on each participant in a controlled rest situation, with HRV features calculated in the time and frequency domains. Significantly lower values of SDNN, pNN50, total power and very low frequency (VLF) power were found in patients with BD. Negative correlations were also found between the scores from the depression questionnaires and a large range of time and frequency domain features using Pearson correlation: SDNN (*r*=−0.415, p=0.016), RMSSD (*r*=−0.347, p=0.048), pNN50 (*r*=−0.436, p=0.011), LF (*r*=−0.379, p=0.03) and HF (*r*=−0.396, p=0.022).

The focus of other studies has been on differences in HRV in bipolar depression compared with healthy controls; work by Chang *et al*^20^ in 2015 compared HRV between these two groups and also between patients with unipolar depression (UD). One hundred and sixteen patients with bipolar depression, 591 physically healthy patients with UD and 421 healthy controls were included in the study, with interviews and self-reported questionnaire scores used as a measure of depression, and 5 min ECG was recorded in lying position after 20 min of rest. Compared with UD, bipolar depression was associated with significantly lower time domain features, along with significantly lower LF and HF power; however, the LF-to-HF ratio was significantly higher in bipolar depression. Comparing the two depressed groups to the healthy controls, features indicative of parasympathetic activity were significantly lower in both groups compared with healthy controls, whereas features supposedly related to sympathetic activity were significantly higher in bipolar depression than in healthy controls, but U participants and healthy controls showed no difference in these features. Basset *et al*^21^ studied 29 BD participants who had been well for at least the last 3 months and compared linear time and frequency domain measures of HRV to 41 participants with major depressive disorder and 38 healthy controls during sleep. The RMSSD of R–R intervals was found to be significantly lower in BD compared with healthy participants, with major depressive disorder participants having significantly reduced HRV compared with healthy controls through the majority of metrics. They suggest ANS dysfunction in BD during sleep through a reduction in parasympathetic activity.

Investigations into differences between HRV in patients with BD in a manic state and healthy controls have also been performed. Henry *et al*^12^ carried out a study on 23 manic BD patients, 14 patients with schizophrenia and 23 healthy controls. HRV was quantified through time domain, frequency domain and non-linear analysis on 5 min of ECG when the participant was at rest. Patients with BD showed lower RMSSD, pNN50 and nHF values and an increase in LF/HF ratio, with indications of reduced parasympathetic activity in patients with BD compared with healthy controls through lower HF power. Reductions in the entropy of the ECG signal were also significant in patients with BD, suggesting reduced HRV. Chang *et al*^22^ also investigated HRV in manic BD patients compared with healthy controls. Sixty-one unmedicated patients with BD and 183 healthy controls had a 5 min section of ECG recorded at rest, and significantly reduced mRR, SDNN, LF power and HF power was found in the manic BD group, suggesting ANS dysregulation in mania.

A study investigating differences in HRV in BD, schizophrenia and healthy controls was performed in 2015 by Quintana *et al*^9^, which included 33 patients with BD, 47 patients with schizophrenia and 212 healthy controls; however, no information is given about the current mental state of the BD group patients. HRV was found to be reduced in both the disorders compared with healthy controls through decreased mean of R–R intervals (mRR) and HF power, and no differences were found between the two disorder groups. These results were also found to be independent of age, body mass index and medication. Moon *et al*,^23^ in 2013, measured HRV features in 41 patients with BD, 35 patients with schizophrenia, 34 patients with post-traumatic stress disorder (PTSD), 34 patients with major depressive disorder and 27 healthy controls, with the aim to discriminate between various mental health disorders using HRV. They found that it was not possible to discriminate between the disorders; however, it was possible to discriminate between the grouped mental health disorders and healthy controls through a reduction in a number of HRV features within the disorders, in particular the HF power component.

Faurholt-Jepsen *et al*^24^ compared HRV measures between 16 BD participants during different affect states. A significant increase in HRV was found during mania compared with both depression and euthymia, with no differences found between euthymia and depression. In addition to these differences, a negative correlation was found between severity of depressive symptoms and HRV. This suggests more severe depression is associated with reductions to HRV, with the opposite direction of correlation found for mania and HRV.

Most of the previous studies on HRV in mental health disorders require a short ECG recording under controlled conditions. The personalised monitoring systems for care in mental health (PSYCHE) project investigated the health of bipolar patients using a wearable monitoring system to record physiological signals, such as HRV, respiration and activity, and a smartphone monitoring systems to determine participant mood and send data to clinicians.^13 25–28^ The project aimed to gain insight into the physiological and mood characteristics of patients with BD over longer periods in a naturalistic setting as opposed to the controlled environments of previous studies. An initial study included eight patients with BD, with over 400 hours of HRV data obtained through the wearable monitoring systems. The data were collected when a patient had been admitted to hospital and was recorded overnight, although no information is given about the length of time used for HRV analysis. Standard time and frequency domain measures were calculated: mRR, SDNN, RMSSD, pNN50, LF, HF and LF/HF ratio from the data, along with sample entropy. The HRV data were then used to classify subjects into one of four mood states (depression, mixed state, hypomania and euthymia) using support vector machines. The initial mood of each patient was assessed by a clinician, with changes in mood monitored by self-reported questionnaires; the mood states were then used to determine the accuracy of the classifiers, which use HRV to predict mood, with an accuracy of around 90%.^28^ No data were collected on healthy controls (or another clinical group), making it difficult to establish the specificity of the findings to BD.

Other studies have investigated the effect therapy and stimulation has on HRV in people with BD. Howells *et al*^29^ studied 12 BD and 9 healthy controls through frequency domain measures of HRV before and after cognitive behavioural therapy. Initially BD participants had elevated HF peaks. After the therapy, there were no differences between BD participants and the controls, suggesting the therapy improves emotional processing of the BD participants. Tanaka *et al*^30^ investigated how stimulation to the wrist affects frequency domain measures of HRV in 25 BD and 22 controls. No differences were found, although hormone levels were different in the groups, suggesting biological background did not influence these changes.

In general, HRV studies including BD participants find that there is a reduction in HRV compared with controls. However, with very few studies meeting all items on the GRAPH checklist (table 3) and with greatly varying methodologies across the studies, it is difficult to summarise the results in any greater detail.

**Table 3:** Table of studies in which HRV measures were calculated on a least one cohort with BD diagnoses. Summary of ECG recordings, HRV measures and results are provided, in addition to their interpretation and adherence to the GRAPH checklist.

Study/year	Cohorts	Data	Parameters	Results	Interpretation	Good Practice Checklist
Bassett *et al*, 21 2016	29 BD, 41 MDD, 38 HC	ECG during sleep, minimum 4 hours	RMSSD, pNN50, SDNN, LF, HF, LF/HF ratio, SD1, SD2	Reduced HRV in BD (RMSSD) and depression (RMSSD, SDNN, SD1 and SD2)	Impaired autonomic function in BD and depression during sleep through a reduction in parasympathetic activity	No ECG sampling rate. No information on R–R interval extraction.
Chang *et al*,^22^ 2014	61 M-BD, 183 HC	5 min ECG at rest	Log transforms of SDNN, VLF, LF, HF power and LF/HF ratio	Significantly reduced mRR, SDNN, LF, HF and LF/HF ratio in BD. Correlations between LF/HF ratio and HF and mania rating scale.	ANS dysregulation is associated with mania in BD through alterations in parasympathetic activity	No information on ECG used, no information on R–R interval cleaning and artefacts.
Chang *et al*,^20^ 2015	116 D-BD, 421 UD, 591 HC	5 min ECG at rest	Log transforms of SDNN, VLF, LF, HF power and LF/HF ratio	Significantly lower SDNN, LF and HF power and higher LF/HF ratio in BD compared with UD. Increased LF power and decreased HF power in BD compared with controls	Sympathetic excitation and parasympathetic impairment in BD compared with controls, with HRV a possible tool to distinguish between UD and BD.	No information on ECG used, no information on R–R interval cleaning and artefacts.
Cohen *et al*,^7^ 2003	39 E-BD, 39 HC	ECG at rest. No length of time given	mRR, SDNN, SDANN, RMSSD, pNN50, VLF, LF, HF power and LF/HF ratio	BD significantly lower RMSSD, total power, nHF and LF/HF ratio	Increase in parasympathetic activity and decrease in sympathetic activity in BD.	No information to rule out psychiatric illness in controls. No information given on the length of the ECG recording. Little information on physiological meaning of HRV parameters until results.
Faurholt-Jepsen *et al*,^24^ 2017	16 BD	Mobile ECG up to 11 days during different affective states	Difference between second longest and second shortest R–R interval every 30 s	Increased variability during manic state compared with euthymia and depression	Autonomic nervous system dysfunction in BD	No ECG sampling rate. No information on R-peak extraction or cleaning
Gruber *et al*,^16^ 2011	23 E-BD, 24 HC	ECG recorded during stimuli	HF power	Increased HF power in BD after stimuli	Increased vagal tone in BD, which is a marker for positive emotion	Little information on recruitment and demographics. Little information on time periods HRV was calculated. Minimal information of R–R interval extraction and cleaning.
Henry *et al*,^12^ 2010	23 M-BD, 14 SZ, 23 HC	5 min ECG at rest	mRR, SDNN, RMSSD, pNN50, LF, HF power and LF/HF ratio	Reduced SDNN in BD, but not significance. Significant increase in LF/HF ratio and decrease in nHF, RMSSD and pNN50 compared with the controls	Decrease in HRV and parasympathetic activity in BD	All items on checklist met
Howells *et al*,^29^ 2013	12 BD, 9 HC	One hour ECG at rest. Before and after cognitive behavioural therapy	Log of LF and HF and peaks in the LF and HF bands	BD increased HF peaks compared with HC. HF peak reduced in BD after therapy	Therapy improved emotional processing in BD as HF peak decreased	All items on checklist met
Lanata *et al*,^26^ 2015	10 BD	Continuous ECG	Sample entropy	Sample entropy is able to estimate long term changes in mental state of BD patients	Sample entropy values of HRV may be able to aid clinicians with diagnosis and management of BD	Little information given on timing of ECG recordings and how R–R intervals are extracted and cleaned. No information on clinical interpretation of sample entropy measures.
Lee *et al*,^19^ 2012	33 SS-BD, 59 HC	5 min ECG at rest	mRR, SDNN, RMSSD, pNN50, VLF, LF, HF power and log total power	Significantly lower SDNN, pNN50, log TP and VLF in BD. Also correlations between symptom severity and HRV parameters were found.	HRV is reduced in BD and may be an effective marker for the disorder	No mention of ECG sampling rate or method of R–R interval extraction or cleaning. Little physiological interpretation of HRV parameters.
Levy *et al,*^18^ 2012	33 E-BD, 22 HC	5 min recording from electrogram	Heart rate	Significantly increased HR parameters in BD	BD patients experience larger changes to ANS on cognitive testing	No information on extraction and cleaning of R–R intervals
Migliorini *et al,*^13^ 2011	1 BD, 8 HC	ECG during four nights	mRR, SDNN, RMSSD, VLF, LF, HF, LF/HF ratio, sample entropy, Lempel-Ziv complexity, detrended fluctuation analysis	Decreased mRR, RMSSD and SDNN in BD. Lempel-Ziv complexity and sample entropy correlated with level of depression	There is dysregulation of the ANS in BD, and HRV is a promising method for measuring mood changes.	No demographic information. All other items on checklist met
Moon *et al*,^23^ 2013	41 BD, 35 SZ, 34 PTSD, 34 UD, 27 HC	5 min ECG at rest	SDNN, RMSSD, VLF, LF, HF, TP, LF/HF ratio and approximate entropy	SDNN, RMSSD, TP, LF and HF all significantly reduced in BD compared with controls	Not possible to use HRV to discriminate between mental health disorders but possible to discriminate from healthy. BD showed most significant HRV changes.	Little participant demographic information. No information on ECG sampling rate or methods to remove artefacts in the R–R interval series
Quintana *et al*,^9^ 2015	33 BD, 47 SZ, 212 HC	5 min of pulse oximetry	HF power	Significant reduction in HF power in BD, independent of age, BMI and medication	Parasympathetic activity is altered in BD, with HRV a possible marker of cardiovascular risk in BD.	Pulse oximeter not ECG, but all other items met on checklist
Tanaka *et al*,^30^ 2013	25 BD, 22 HC	No information on ECG	LF, HF and LF/HF ratio	No difference between BD and HC after stimulation to wrist	No difference in HRV between groups, biological background did not influence hormonal reaction observed.	No details of collection method of ECG. No information on R–R interval extraction and cleaning
Todder *et al*,^15^ 2005	39 E-BD, 39 HC	ECG at rest. No length of time given	Non-linear parameters	No significant differences between any parameters	There is no disturbance in the ANS	No information to rule out psychiatric illness in controls. No information given on the length of the ECG recording. Little information on physiological meaning of HRV parameters until results.
Valenza *et al*,^25^ 2015	8 BD	ECG recorded approximately 10 min during tasks	Point-process-based non-linear autoregressive integrative model	Around 90% accuracy of predicting depression or euthymia in BD.	A link between ANS function and BD exists, with parameters measuring ANS able to predict mood or emotion of patients	Little information on method of ECG recording and extraction and cleaning of R–R intervals
Voggt *et al*,^17^ 2015	90 E-BD, 62 HC	30 min ECG recording	SDNN, LF, HF and LF/HF ratio	Significantly lower SDNN, LF and HF in BD	SDNN may be used to study interventions to reduce cardiovascular disease in BD	Most items met, no mention of ECG sampling rate or causes of artefacts

ANS, autonomic nervous system; BD, bipolar disorder; BMI, body mass index; D-BD, depressed bipolar disorder; E-BD, euthymic bipolar disorder; GRAPH, Guidelines for Reporting Articles on Psychiatry and Heart rate variability; HC, healthy control; HF, high frequency power; HRV, heart rate variability; LF, low frequency power; LF/HF ratio, low frequency to high frequency ratio; M-BD, manic bipolar disorder; MDD, major depresive disorder; mRR, mean of R–R interval; pNN50, perentage of R-R intervals over 50ms; PTSD, post-traumatic stress disorder; RMSSD, root mean square of successive differences; SDANN, SD of average R–R intervals; SDNN, SD of R–R intervals; SD1, standard deviation in Poincare plot y=-x direction; SD2, standard deviation in Poincare plot y=x direction; SS-BD, subsyndromal depression bipolar disorder; SZ, schizophrenia; TP, total power; UD, unipolar depression; VLF, very low frequency.

### HRV in BPD

Few studies have explored HRV in BPD. A study carried out by Austin *et al* investigated respiratory sinus arrhythmia (RSA) in nine patients with BPD and 11 healthy controls. Variations in R–R intervals due to respiration is known as RSA and is a measure of synchronicity of HRV and respiration rate and considered a marker of parasympathetic nervous system activity. The study showed significant difference in parasympathetic activity between patients with BPD and healthy controls through differences in RSA.^31^ Ebner-Priemer *et al* recorded 24 hours ECG signals on 50 patients with BPD and 50 healthy controls.^32^ HRV was calculated for the period at night at which the average HR was lowest; the results from the study tested the hypothesis that HRV is lower in BPD. However, the HF components of the HRV were found to be higher in patients with BPD, which is surprising as HF activity is related to parasympathetic activity. Furthermore, a study investigating parasympathetic and sympathetic activity through the use of RSA in 12 patients with BPD and 28 healthy controls had ECG recorded for three 5 min stages (at rest or stressed) found BPD was associated with lower values of RSA suggesting increased levels of sympathetic activity and decreased levels of parasympathetic activity.^33^ Meyer *et al* recorded 5 min ECG signals on 27 participants with BPD, 23 in remission from BPD, 18 suffering from PTSD and 23 healthy controls.^34^ Significant differences were only found between PTSD participants and controls; however, BPD participants had reduced variability across linear time and frequency domain measures compared with controls.

The lack of studies in people with BPD makes it difficult to draw conclusions about how HRV is altered in the disorder, especially as only two of the four studies included here use any of the standard HRV measures. Although these studies follow the GRAPH checklist relatively well (table 4), Ebner-Priemer and Meyer use ECG recordings over greatly varying timescales with differing results, with Meyer finding no differences between groups and Ebner-Premier finding increased parasympathetic activity in BPD, opposite to the expected result. More studies involving BPD participants are required which closely follow the GRAPH checklist before we can speculate how HRV is altered in BPD.

**Table 4:** Table of studies in which HRV measures were calculated on a least one cohort with BPD diagnoses. Summary of ECG recordings, HRV measures and results are provided, in addition to their interpretation and adherence to the GRAPH checklist.

Study/year	Cohorts	Data	Parameters	Results	Interpretation	Good practice checklist
Austin *et al*,^31^ 2007	9 BPD, 11 HC	Four times 10 min ECG watching films	RSA	Significantly reduced RSA in BPD	Lower RSA is linked to reduced parasympathetic activity in BPD, with changes in RSA much less after emotional stimuli	Little demographic information. HRV not calculated
Ebner-Priemer *et al*,^32^ 2007	50 BPD, 50 HC	24 hours ambulatory ECG	mRR, HF power, HF power at night	Increased mRR and HF power in BPD	Increased HF power indicates increased parasympathetic activity, opposite to the expected findings	Little demographic information. No information on R–R interval extraction, cleaning and dealing with artefacts
Meyer *et al*,^34^ 2016	27 BPD, 23 BPD in remission, 18 PTSD, 23 HC	5 min at rest. After emotional face classification	RMSSD, SDNN, NN50, total power, LF, HF and LF/HF ratio	HRV lower in all groups compared with HC. Only significant for PTSD	No difference in HRV between BPD and HC. This may differ at varying stress levels	No ECG sampling frequency. R–R interval extraction and cleaning using Kubios, with no further detail
Weinberg *et al*,^33^ 2009	12 BPD, 28 HC	Three times 5 min ECG	RSA	Decreased RSA values in BPD	Increased levels of sympathetic activity and decreased levels of parasympathetic activity indicated by reduced RSA	No information on disease characteristics and little demographic information. HRV not calculated.

BPD, borderline personality disorder; HC, healthy control; HF, high frequency; HRV, heart rate variability; LF, low frequency; LF/HF ratio, low frequency to high frequency ratio; NN50, number of R-R intervals over 50ms; PTSD, post-traumatic stress disorder; RMSSD, root mean square of successive differences; RSA, respiratory sinus arrhythmia; SDNN, SD of R–R intervals.

There have been no published studies where BPD and BD have been directly compared, making any conclusions tentative at best. The heterogeneity of methodologies employed in these studies adds a further level of complexity to any comparisons. At present there is insufficient evidence to allow comparison of HRV in BPD and BD to be made.

## Conclusion

HRV is an important physiological marker in psychiatric illness and may provide important information about underlying phenotypes as well as cast light on the increased cardiovascular risk associated with psychiatric disorder. At present there is little consensus with respect to methodology that makes comparison difficult. The majority of previous studies on HRV in BD and BPD fail to meet every item on the GRAPH checklist. Interpretation of previous findings is difficult as there is often a lack of information available to accurately reproduce and compare results or build on previous work. If future studies were to closely follow this set of guidelines, it may accelerate HRV research in mental health and aid interpretation and reproducibility.

Given that diurnal rhythms disturbance is inherent in many psychiatric disorders and mobile ECG monitoring can now record signals for days at a time, diurnal patterns of HR and HRV measures may provide further insight into nervous system function. Future studies may investigate how sleep–wake cycles are linked to HRV and whether psychiatric disorders are associated with altered diurnal patterns of HRV.
